# The Roles of Lpar1 in Central Nervous System Disorders and Diseases

**DOI:** 10.3389/fnins.2021.710473

**Published:** 2021-07-27

**Authors:** Dongqiong Xiao, Xiaojuan Su, Hu Gao, Xihong Li, Yi Qu

**Affiliations:** ^1^Key Laboratory of Birth Defects and Related Diseases of Women and Children, Department of Pediatrics, Ministry of Education, West China Second University Hospital, Sichuan University, Chengdu, China; ^2^Key Laboratory of Birth Defects and Related Diseases of Women and Children, Department of Emergency, Ministry of Education, West China Second University Hospital, Sichuan University, Chengdu, China

**Keywords:** Lpar1, Edg2, astroglia, microglia, oligodendrocyte, astrocyte

## Abstract

Lysophosphatidic acid receptor 1 *(Lpar1)*, which is found in almost all human tissues but is most abundant in the brain, can couple to G protein-coupled receptors (GPCRs) and participate in regulating cell proliferation, migration, survival, and apoptosis. Endothelial differentiation gene-2 receptor (Edg2), the protein encoded by the *Lpar1* gene, is present on various cell types in the central nervous system (CNS), such as neural stem cells (NSCs), oligodendrocytes, neurons, astrocytes, and microglia. *Lpar1* deletion causes neurodevelopmental disorders and CNS diseases, such as brain cancer, neuropsychiatric disorders, demyelination diseases, and neuropathic pain. Here, we summarize the possible roles and mechanisms of *Lpar1/*Edg2 in CNS disorders and diseases and propose that *Lpar1/*Edg2 might be a potential therapeutic target for CNS disorders and diseases.

## Introduction

**FIGURE 1:**
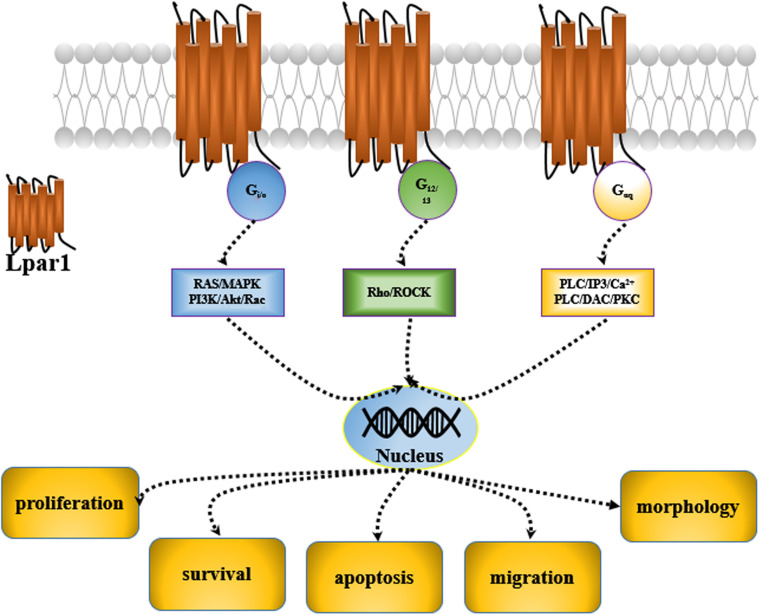
LPA signaling causes G proteins to play different roles in the CNS.

Lysophosphatidic acid receptor 1 (*Lpar1*) was first discovered in the developing brain in 1996 (Hecht et al., 1996). It was found to be enriched in the ventricular zone (VZ) of the embryonic cerebral cortex. Then, five other receptors of the *Lpar* family, including *Lpar2, Lpar3, Lpar4, Lpar5*, and *Lpar6*, were characterized, and they were all found to be expressed in the central nervous system (CNS) (Choi et al., 2010). These receptors are cell surface G protein-coupled receptors (GPCRs), and their ligand is lysophosphatidic acid 1-acyl-sn-glycerol-3-phosphate (LPA). Many potential roles for LPA and LPA receptor signaling have been reported; LPA and LPA receptors are mediated by second messenger pathways after coupling to the G proteins, Gi/0, Gαq, and G12/13 and participate in the regulation of cell proliferation, migration, survival, apoptosis, and morphology (Panetti, 2002; Takuwa et al., 2002; de San Roman et al., 2015; Figure 1).

## Characteristics of Edg2

**FIGURE 2:**
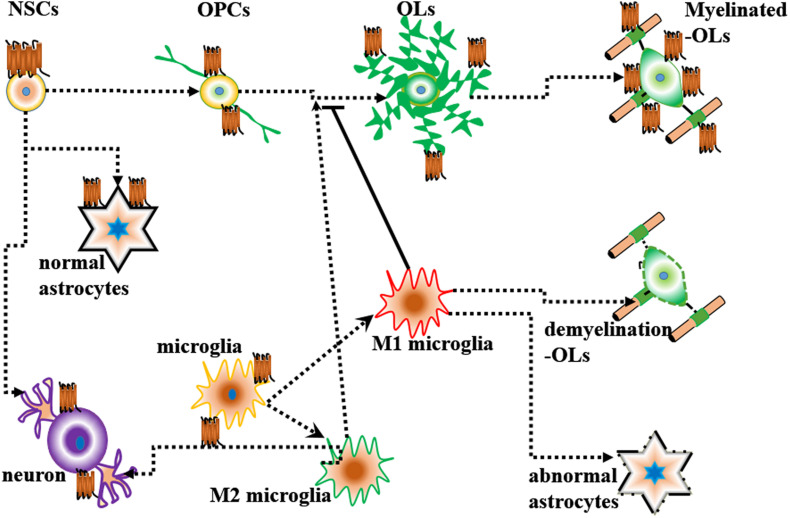
Lpar1/Edg2 is present on various cell types and interacts with glial cells and neurons.

**FIGURE 3:**
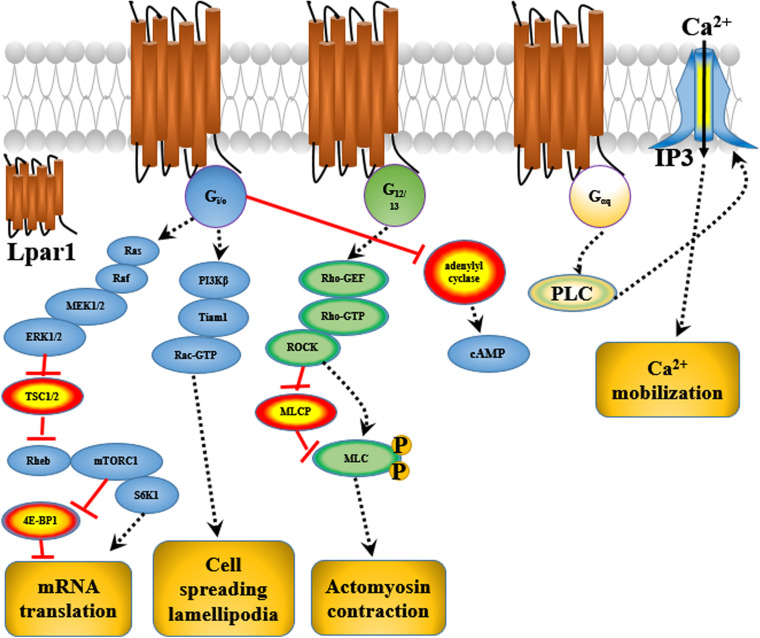
The possible mechanisms by which Lpar1 exerts its effects in glial and neuronal cells.

Endothelial differentiation gene-2 receptor (Edg2) is encoded by *Lpar1*, which is found in almost all human tissues but is most abundant in the brain (An et al., 1997). Human Edg2 is a 364-amino acid protein with a putative nuclear translocation signal, an N-terminal acidic domain, and a cysteine-rich C-terminal domain containing a putative zinc finger structure (Hla et al., 1995; An et al., 1997; Allard et al., 1999). The polypeptide sequence of Edg2 was highly conserved during evolution, suggesting that it may be an important regulator of general nuclear function (Hla et al., 1995). Edg2 is a seven-transmembrane domain receptor with high specificity for LPA (Erickson et al., 1998). Edg2 is present on various cell types, such as astrocytes, oligodendrocytes, microglia, and neurons, in the CNS (Allard et al., 1999; Yung et al., 2015; Figure 2). Edg2 can induce cellular proliferation and morphological changes. It has been proposed to be involved in many physiological and pathological processes, including neurogenesis, myelination, angiogenesis, wound healing, and cancer progression (Allard et al., 1999; Contos J.J. et al., 2000; Yung et al., 2015). Edg2 can efficiently couple to the yeast heterotrimeric G-protein in response to LPA binding and activate the yeast mitogen-activated protein kinase (MAPK) pathway (Erickson et al., 1998; Choi et al., 2010). Edg2 can also couple with Gi/o to activate Ras and then the MEK-ERK pathway. Subsequently, ERK acts to inhibit the tuberous sclerosis complex (TSC1/2), thereby increasing GTP loading on Ras homolog enriched in brain (Rheb) and thus activating mTORC1. mTORC1 then phosphorylates downstream targets, such as S6K1 and eukaryotic initiation factor 4E-binding protein (4E-BP1), ultimately leading to increased mRNA translation (Winter et al., 2010; Figure 3). Lpar1 with the P308S, I310T, and Y311H mutations might not interact with helix 8, which corresponds to codons 315–326 of Lpar1, leading to structural defects and retention of Edg2 in the endoplasmic reticulum (ER). In addition, mutation of Lpar1 can alter its intracellular activities, such as Ca^2+^ mobilization, inhibition of cAMP formation, and cytoskeletal changes, which are mainly mediated by Gq, Gi/o, and G12/13, respectively (Ishii et al., 2017). Lpar1 deletion can cause neurodevelopmental disorders and CNS diseases, including brain cancer, neuropsychiatric disorders, demyelination diseases, and neuropathic pain (Table 1).

**TABLE 1:** The role of Lpar1 in the CNS.

Cell type	Disease model	Function	Mechanism	References
*NSCs*	Neurological and psychiatric diseases	Progenitor differentiation into intermediate progenitor cells	Cell division	McDonald et al., 2020
	NA	LPA-*Lpar1* signaling enhance neurogenesis and decrease programmed cell death	Cells averting programmed cell death, through premature cell cycle exit and neuronal differentiation	Kingsbury et al., 2003; McDonald et al., 2020
*Oligodendrocytes*	NA	Oligodendrocytes morphology	Cytoskeletal rearrangements, which are involved in oligodendrocytes outgrowth to membrane sheath formation	Nogaroli et al., 2009
*OPCs*	A model precursor OL line	Cell migration	LPA/Lpar1/Rho-ROCK	Dawson et al., 2003
	Immortalized oligodendrocyte cell line (OLP6)	Anti-apoptosis and promote OPCs differentiation	Lpar1/Gi/o/MAPK; Lpar1/Gi/o/adenylyl cyclase inhibition	Matsushita et al., 2005
*Pre-OLs*	NA	Latest stages of oligodendrocyte maturation	ERK1/ERK2	Stankoff et al., 2002
	NA	Latest stages of oligodendrocyte maturation		Nogaroli et al., 2009
*Mature OLs*	Demyelinating diseases	Trafficking of PLP	Vesicle-mediated endocytosis and recycling to the plasma membrane disrupt	Garcia-Diaz et al., 2015
	**/**	Stress-induced OLs apoptosis		Garcia-Diaz et al., 2015
		Enhance oligodendrocytes differentiation and myelination		
	HIV infected patients WMI	/	ATX-LPA signaling	Wheeler et al., 2016
	Ischemic reperfusion injury	Anti-apoptosis	/	Savitz et al., 2006
*Astrocytes*	Traumatic brain injury	Reactive astrocytes	/	
	Depression	Astrocytes DNA synthesis	Lpar1/ERK	Nakajima et al., 2018
	Depression	Astrocytes DNA synthesis	Lpar1/Gi/o/Src family tyrosine kinase/MMP-9	Abe et al., 2019
	Astrocytic shape changes	Mitogenic activity	LPA/Lpar1/NGF	Tabuchi et al., 2000
		Astrocytes DNA synthesis	ERK1/2/GDNF	Kajitani et al., 2016
*Neuron*	Neuroblastoma cells	Cell migration	Lpar1/Gi/o/Mmp-2	Kato et al., 2012
	Neuropathic allodynia	Neuronal reorganization through Abeta (Aβ)-fibber	Lpar1/ERK	Xie et al., 2008
	Hippocampal neurons	Decrease programmed cell death and enhance neurogenesis	Premature cell cycle exit and neuronal differentiation	Kingsbury et al., 2003
		Increase neuronal excitability	PLC-dependent signaling pathway and voltage-gated potassium (Kv) channel/Lpar1/Ca2^+^ current	Park et al., 2015
	Neuroblast cells	Represses Lpar1 promoter activity	HEB binding to EB3 inhibit Lpar1 promoter	Kim et al., 2019
	Neuroblastoma cells	Cell motility	Lpar1 coupled to Gi, Gq, G12/13, and ROCK	Hayashi et al., 2012
	Neuropathic pain mice	/	Lpar1/Edg2 mediated amplification of spinal LPA production	Uchida et al., 2014
	Neuropathic pain	Development of allodynia and hyperalgesia	LPA/Lpar1/Rho-ROCK	Inoue et al., 2004
	Neuropathic pain	Upregulation of Lpar1/Edg2 and Nav1.8 in DRGs of normal rats	LPA/Lpar1/G protein/MAPK/PKC/Rho	Pan et al., 2016
	Alzheimer’s disease (AD)	Circ-Lpar1 might regulate neuronal cell death and neuroinflammation by sponging their target miRNAs	/	Li et al., 2020
*Microglia*		Microglia migration	Rho and ERK1/2	Bernhart et al., 2010
	Neuropathic pain	Microglia activation	Lpar1/microglia activation, ATP release and BDNF upregulation	Fujita et al., 2008
		LPA activated pro-inflammatory process and triggers M1 polarization	LPA/Lpar1	Fransson et al., 2020
		Alleviate demyelination and microglia polarization	Lpar1/MAPK/Keap1/Nrf2	Choi et al., 2020
		Neurotoxic microglia polarization	Lpar1/MAPK	Plastira et al., 2020
		Activating microglia induced demyelination	LPA/Lpar1	Santos-Nogueira et al., 2015

## Distribution of Edg2

Information about Edg2 distribution was obtained from in situ hybridization studies. Edg2 is expressed in most mammalian cells and tissues. In the brain, Edg2 appears to be localized within specific brain regions and in certain cell types.

In the rodent embryonic cortex, *Lpar1* mRNA is predominately present in the neurogenic VZ (Hecht et al., 1996; Allard et al., 1998). *Lpar1* mRNA is first detected on postnatal day 2 (P2) in the medulla oblongata and cervical spinal cord and is observed in the deep white matter of the cerebellum and brainstem on postnatal day 14 (P14) (Stankoff et al., 2002). However, in adults, Edg2 receptor mRNAs are restricted to white matter tracts (Handford et al., 2001; Stankoff et al., 2002). This phenomenon was also reported in another study. Functional [^35^S]GTPγS autoradiography revealed that *Lpar1*/Edg2 binding sites were observed in myelinated areas of the white matter, such as the corpus callosum, internal capsule and cerebellum, in the brains of adult rodent and humans (de San Roman et al., 2015). Many cells in the brain, such as neural stem cells (NSCs), oligodendrocytes, neurons, astrocytes, and microglia, can express *Lpar1*/Edg2.

## Functions of Edg2

### Edg2 in NSCs

Neural stem cells are multipotent cells that can generate radial glia, oligodendrocyte progenitor cells (OPCs), neurons, and astrocytes (Urbán et al., 2019). In rodents, NSCs are found in the sub-granular zone (SGZ) of the dentate gyrus (DG) in the hippocampus and ventricular-sub-ventricular zone (V-SVZ) lining the lateral ventricles (Yao et al., 2012; Urbán et al., 2019). NSC maintenance, division, and differentiation are controlled by multiple genes and steps. Any alteration in the program that regulates genes may disrupt NSC maintenance, division, and differentiation.

LPA-*Lpar1* signaling may enhance NSC differentiation. *Lpar1*/Edg2 is expressed on embryonic NSCs. LPA-*Lpar1* signaling increases non-vertical cleavage of apical progenitor cells and enhances the differentiation of progenitors into intermediate progenitor cells (McDonald et al., 2020).

McDonald et al. (2020) reported that LPA-*Lpar1* signaling influences NSC cleavage plane orientation and early cell fate. LPA-*Lpar1* signaling alters NSC cleavage plane orientation by disrupting cellular adherens junctions (AJs), leading to premature neurogenesis. Enhanced LPA-*Lpar1* signaling has been shown to enhance neurogenesis and decrease programmed cell death. Intermediate neural progenitor cells (NPCs) expressing T-box brain protein 2 (Tbr2) are typically localized to the cortical plate during early to mid-neurogenesis. LPA-*Lpar1* signaling increases the number of Tbr2^+^ cells, suggesting that LPA-*Lpar1* signaling-induced cleavage plane abnormalities may trigger an increase in the Tbr2^+^ cell number. An increase in the number of Tbr2^+^ cells indicates aversion of programmed cell death through premature cell cycle exit and neuronal differentiation (Kingsbury et al., 2003; McDonald et al., 2020).

### Edg2 in Oligodendrocytes

**FIGURE 4:**
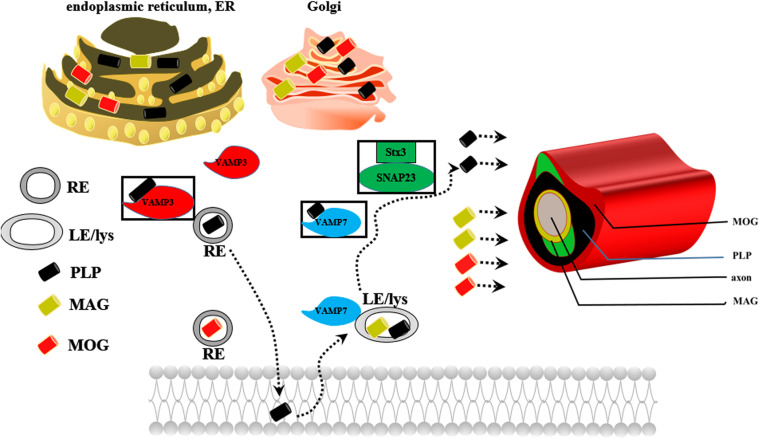
The possible mechanism underlying the role of PLP in myelin biogenesis. REs, recycling endosomes; LEs/Lys, late endosomes/lysosomes.

Oligodendrocytes are myelin-forming cells in the CNS. Myelin sheaths are formed by oligodendrocytes, which wrap axons during late fetal and early postnatal stages (Piaton et al., 2010). Differentiation of NSCs into oligodendrocytes occurs in multiple stages and is controlled by differentiation-regulated transcription factors, which are stage-specific markers (White and Kraemer-Albers, 2014; Czepiel et al., 2015; Goldman and Kuypers, 2015). During development, mature oligodendrocytes originate from NSCs that are generated in discrete areas of the CNS and migrate to their destination to differentiate into post-migratory oligodendrocytes, OPCs, pre-myelinating oligodendrocytes (pre-OLs) and mature oligodendrocytes in a cell autonomous fashion (Bradl and Lassmann, 2010; Czepiel et al., 2015; Domingues et al., 2016). A key step controlling whether OPCs/pre-OLs adopt a myelinating fate is specific axon-glia recognition (White and Kraemer-Albers, 2014). Myelin biogenesis requires intricate membrane sorting and trafficking machinery following axon recognition and positioning of myelination-competent oligodendrocytes as well as directed vesicular trafficking pathways. Proteolipid protein (PLP) mRNA is translated in the rough endoplasmic reticulum (RER) of the cell body, packed into vesicles in the Golgi apparatus (GA), and transported to peripheral myelin (Bauer et al., 2002). PLP endocytosis and recycling to the plasma membrane are regulated by soluble N-ethylmaleimide-sensitive factor attachment protein receptors (SNAREs); PLP is recycled by recycling endosomes (REs) via a VAMP3-dependent pathway and by late endosomes (LEs)/lysosomes (Lys) via a VAMP7-dependent pathway (Feldmann et al., 2011; White and Kraemer-Albers, 2014; Figure 4). Impaired trafficking of PLP from the ER and GA to the plasma membrane leads to oligodendrocyte apoptosis and demyelination (Bauer et al., 2002; Feldmann et al., 2011).

The *Lpar1*/Edg2 receptor is expressed in OPCs and mature oligodendrocytes in the postnatal rat brain (Nogaroli et al., 2009; Garcia-Diaz et al., 2015). *Lpar1*/Edg2, which is expressed on the oligodendrocyte cell membrane and oligodendrocyte processes and co-localizes with myelin basic protein (MBP), plays crucial roles in regulating oligodendrocyte migration, survival, apoptosis, morphology, and myelination.

First, *Lpar1*/Edg2 can regulate OPC migration. LPA-*Lpar1* signaling activates downstream Rho-ROCK and stimulates actomyosin contraction in OPCs, thus modulating OPC migration to specific regions of the brain (Dawson et al., 2003).

Second, *Lpar1*/Edg2 can regulate oligodendrocyte survival and apoptosis. Matsushita et al. (2005) found that *Lpar1*/Edg2 is expressed in the immortalized oligodendrocyte cell line OLP6. OLP6 cell apoptosis was shown to be induced upon serum withdrawal, and this apoptosis was shown to be inhibited by the addition of 5% serum or 10 μM LPA, a *Lpar1*/Edg2 ligand, to the serum-free medium. These results indicate that the activation of LPA-*Lpar1* signaling plays an anti-apoptotic role in oligodendrocytes, enhancing the survival of immature oligodendrocytes.

Third, *Lpar1*/Edg2 can regulate oligodendrocyte morphology. *Lpar1*/Edg2 was detected in O4-positive cells with processes with both simple and complex morphologies (Nogaroli et al., 2009). The effect of LPA-*Lpar1* on oligodendrocyte morphology may be associated with cytoskeletal rearrangements, which are involved in the outgrowth of oligodendrocytes for membrane sheath formation (Nogaroli et al., 2009). The absence of *Lpar1*/Edg2 leads to fiber disorganization and alterations in morphology via an increase in the number of unmyelinated axons and the myelin g-ratio (Garcia-Diaz et al., 2015).

Finally, *Lpar1*/Edg2 can enhance oligodendrocyte differentiation and myelination. Matsushita et al. (2005) found that when LPA binds to *Lpar1*/Edg2 expressed in immortalized oligodendrocyte cells (OLP6 cells), Gi/o is activated, leading to phosphorylation of p44/p42 MAPK, inhibition of adenylyl cyclase, thus promotion of the differentiation of OPCs into immature OLs and then into mature OLs. Stankoff et al. (2002) observed that *Lpar1*/Edg2 is selectively expressed in mature post mitotic oligodendrocytes prior to myelination, contributes to the latest stages of oligodendrocyte maturation, and may be involved in myelination. LPA increases the number of cells positive for MBP but not myelin oligodendrocyte glycoprotein (MOG), which implies that LPA may play a crucial role in regulating the later stages of oligodendrocyte maturation (Nogaroli et al., 2009). Myelin biogenesis involves translation of PLP mRNA in the RER, sorting of PLP components in the *trans-*GA, and subsequent transport of these components to the plasma membrane by endocytic recycling. Dysfunction of any of these processes leads to retention of PLP in the RER or GA and initiation of myelin diseases characterized by developmental hypomyelination and demyelination. Indeed, Lpar1 activation has been associated with vesicle-mediated endocytosis (Nogaroli et al., 2009), which participates in PLP trafficking, oligodendrocyte maturation and myelination. A lack of the Lpar1 receptor results in accumulation of myelin PLP in the RER, causes PLP protein to be trapped within the RER, and causes anomalous trafficking of PLP, suggesting stress-induced apoptosis of oligodendrocytes and disruption of PLP endocytosis and recycling to the plasma membrane (Garcia-Diaz et al., 2015). We speculate that *Lpar1*/Edg2 may participate in PLP trafficking, thus leading to oligodendrocyte maturation and later myelination.

### Edg2 in Neurons

Cortical neurogenesis, during which NPCs/NSCs differentiate into glial progenitors and young post-mitotic neurons, occurs from E10-18 in mice. Mature neurons establish polarity through neurite specification and develop axons and dendrites (Yung et al., 2015). The interaction between axons and glia is programmed, and disorders affecting this interaction may lead to demyelinating diseases or neuropathic pain.

Lysophosphatidic acid receptor 1/Endothelial differentiation gene-2 receptor is expressed on dorsal root ganglion (DRG) neurons (Pan et al., 2016) and hippocampal neurons (Park et al., 2015), which regulates neuronal morphology, motility, growth cone collapse, calcium signaling, and proliferation. LPA has been reported to induce necrosis and apoptosis of hippocampal neurons (Park et al., 2015). *Lpar1*/Edg2 activation can increase the excitability of hippocampal neurons. This increase in neuronal excitability seems to be associated with gintonin-induced *Lpar1*/Edg2 activation via the phospholipase C (PLC)-dependent signaling pathway and voltage-gated potassium (Kv) channels (Park et al., 2015). Gintonin-mediated *Lpar1*/Edg2 activation is associated with N-methyl-D-aspartic acid (NMDA) receptors and consequently increased synaptic transmission (Park et al., 2015). Gintonin increases the transient Ca^2+^ current through *Lpar1*/Edg2 activation (Park et al., 2015). The neuroprotective molecular mechanisms of gintonin are mediated through multiple processes, including anti-oxidative stress, an increase in brain-derived neurotrophic factor (BDNF) and Sirt1 expression, and anti-apoptotic/inflammatory effects after *Lpar1*/Edg2 activation (Nam et al., 2020). *Lpar1*/ERK contributes to neuronal reorganization through Abeta (Aβ) fibers, and leads to neuropathic allodynia (Xie et al., 2008). Neuronal damage is significantly reduced in hyperbaric oxygen (HBO)-treated rats compared with control rats after hypoxic-ischemic brain injury, and *Lpar1*/Edg2 plays a neuroprotective role in the brains of HBO-treated rats (Hirata et al., 2007). Enhanced LPA-*Lpar1* signaling has been shown to inhibit programmed cell death and enhance neurogenesis through premature cell cycle exit and neuronal differentiation (Kingsbury et al., 2003).

**FIGURE 5:**
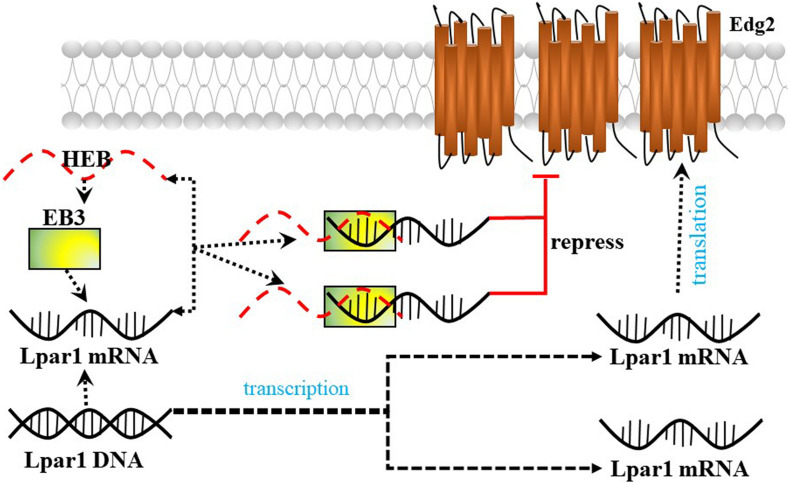
The possible mechanism by which HEB repress Lpar1.

Lysophosphatidic acid receptor 1/Endothelial differentiation gene-2 receptor is also found in developing neurons (neuroblasts) and enhances the differentiation of neocortical neuroblasts into mature neurons. Kim et al. (2019) reported that the E-protein HeLa E-box binding protein (HEB) (encoded by the *Tcf12* gene) represses *Lpar1*/Edg2 promoter activity by binding to the third E-box (EB3) in mouse neocortical neuroblasts and plays an important role in brain development (Figure 5).

### Edg2 in Astrocytes

Astrocytes account for approximately 20–40% of all glial cells in the CNS (Ji et al., 2019). Astrocytes originate from NPCs and are classified into two types: type 1 astrocytes (protoplasmic astrocytes, localized in the gray matter) and type 2 astrocytes (fibrous astrocytes, localized in the white matter) (Domingues et al., 2016). Astrocytes provide structural and nutrient support for neurons and play an important role in many critical neural processes, such as by contacting inter-neuronal synapses, regulating neuroinflammation, and thus mediating CNS diseases, including gliomas and neuropsychiatric, neurodegenerative and neurodevelopmental diseases (Tabuchi et al., 2000). Astrocytes are quiescent in normal resting CNS tissue. However, astrocytes become activated by many mechanisms upon injury, resulting in mild astrogliosis (Domingues et al., 2016).

Lysophosphatidic acid receptor 1/Endothelial differentiation gene-2 receptor enhances astrocytes proliferation. *Lpar1*/Edg2 is highly expressed in astrocytes (Tabuchi et al., 2000; Nakajima et al., 2018). LPA-*Lpar1* has mitogenic activity in astrocytes. LPA stimulates DNA synthesis in rat astrocytes (Tabuchi et al., 2000). *Lpar1*/Edg2 contributes to 2-O-carba-cyclic phosphatidic acid (2ccPA)-induced DNA synthesis in astrocytes through the activation of the ERK pathway (Nakajima et al., 2018). LPA-*Lpar1* exerts mitogenic activity in astrocytes and induces the expression of several genes, including nerve growth factor (NGF) (Tabuchi et al., 2000).

Lysophosphatidic acid receptor 1/Endothelial differentiation gene-2 receptor can also regulate astrocyte morphology and thus influence synaptic transmission and plasticity (Tabuchi et al., 2000). LPA has a dramatic effect on astrocyte morphology, potentially altering the volume of the synaptic cleft, and thus modulate neurotransmitter concentrations and interactions between neurons and glia. Tabuchi et al. (2000) found that LPA-*Lpar1* induces Ca^2+^ mobilization in rat primary astrocytes, which indicates that LPA may also serve as a neuromodulator.

In addition, *Lpar1*/Edg2 can regulate the secretion of neurotrophic factor genes by astrocytes. LPA-*Lpar1* signaling increases glial cell line-derived neurotrophic factor (GDNF) mRNA expression in astrocytes. GDNF is a neurotrophic/growth factor that supports neurogenesis, gliogenesis, neural plasticity and cell survival in the brain (Abe et al., 2019). GDNF expression has been found to be decreased in patients with major depression (Abe et al., 2019), and antidepressant treatment can significantly increase GDNF levels in patients with major depression (Abe et al., 2019). Fibroblast growth factor receptor (FGFR), FGFR substrate 2α (FRS2α) and ERK1/2 are related to GDNF expression through the Lpar1-mediated signaling pathway (Kajitani et al., 2016). Gi/o-coupled Lpar1-induced phosphorylation of ERK1/2 in astrocytes mediates GDNF mRNA expression (Kajitani et al., 2016). The antidepressant amitriptyline increases GDNF production via the Gi/o-coupled Lpar1/matrix metalloproteinase-9 (MMP-9)/FGFR/ERK cascade. Recent findings indicate that amitriptyline activates a Lpar1/Gi/o/Src family tyrosine kinase/MMP-9 cascade in astroglial cells (Abe et al., 2019).

LPA-*Lpar1* promotes astrocytes to transcribe various genes and secrete a variety of cytokines, such as immediate-early genes (*c-fos* and *c-jun*, which promote cell proliferation and differentiation), NGF, IL-1, IL-3, and IL-6, the levels of which are increased when astrocytes are activated or proliferate (Tabuchi et al., 2000).

### Edg2 in Microglia

Microglia, which arise from hematopoietic stem cells in the yolk sac during early embryogenesis, account for approximately 10% of all glial cells in the CNS (Domingues et al., 2016). Microglia can be classified into two groups according to phenotype: M1 microglia (pro-inflammatory and neurotoxic) and M2 microglia (anti-inflammatory microglia involved in neural repair) (Yu et al., 2020). Steady-state microglia might promote oligodendrocyte differentiation by providing trophic support to OPCs. However, LPS-mediated inflammation polarizes microglia, thus preventing oligodendrocyte differentiation (Domingues et al., 2016). Microglia promote apoptosis of differentiated cells via mechanisms including tumor necrosis factor (TNF) signaling, NGF secretion, and reactive oxygen species (ROS) production (Thion et al., 2018). Microglia participate in the establishment of neuronal connections by promoting outgrowth or fasciculation of axonal tracts during pre- and post-natal development. Microglia can also influence neuronal migratory processes. Additionally, microglia are involved in promoting the formation of excitatory synapses (Bartels et al., 2020).

Lysophosphatidic acid receptor 1/Endothelial differentiation gene-2 receptor has been found to be expressed in mouse and rat microglia and to be related to Ca^2+^ influx (Möller et al., 2001). LPA-*Lpar1* might activate microglia in response to CNS injury (Möller et al., 2001). Human immortalized C13NJ microglia express Lpar1 (Bernhart et al., 2010). *Lpar1/*Edg2 activation leads to the activation of Rho and ERKs, which triggers an increase in ATP production, alters the actin and tubulin cytoskeleton, and decreases cell migration (Bernhart et al., 2010). Neuroinflammation is critical during neurodegeneration. LPA-*Lpar1* signaling can induce microglial polarization toward a protective or neurotoxic phenotype by activating MAPK (Plastira et al., 2020). Santos-Nogueira et al. (2015) found that LPA levels are increased in the mouse spinal cord after contusion injury. Exogenous LPA injection was shown to activate microglia and induce demyelination secondary to axon or myelin damage. Microglial Lpar1 is responsible for exogenous LPA-induced demyelination. These findings indicate that LPA leads to demyelination by activating microglia Lpar1 (Santos-Nogueira et al., 2015).

## The Roles of Lpar1 in CNS Disorders and Diseases

### Brain Tumors

Matrix metalloproteinases (MMPs) are involved in extracellular matrix degradation and associated with tumor invasion and metastasis. Mutated *Lpar1* stimulates the expression and activity of MMP-2 in rat neuroblastoma cells (Kato et al., 2012). It has been reported that mutated *Lpar1* increases cell motility and invasion of rat neuroblastoma cells (Hayashi et al., 2012). These results suggest that *Lpar1/*Edg2 might inhibit the motility and invasion of neuroblastoma cells. Hirane et al. (2013) reported that *Lpar1* markedly suppresses B103 cell motility stimulated by hydrogen peroxide and 2,3-dimethoxy-1,4-naphthoquinone (DMNQ). It has been found that *Lpar1/*Edg2 plays different roles in cell proliferation and the migration of rat neuroblastoma cells (Hayashi et al., 2012). *Lpar1/*Edg2 couples to Gi, Gq, G12/13, and ROCK to affect cell motility (Hayashi et al., 2012). *Lpar1/*Edg2 receptor couples to Gα12/13 to mediate Rho-GEF/RhoA-GTP and thus affect actomyosin contraction and couples to Gi to mediate PI3Kβ/Tiam1/Rac-GTP and therefore affect cell spreading and lamellipodia formation, thus altering the migration and motility of B103 neuroblastoma cells (Van Leeuwen et al., 2003; Figure 3). Loss of primary cilia has been shown to affect cell proliferation in glioblastoma. *Lpar1/*Edg2 accumulates in primary cilia and has been reported to be responsible for the proliferation of glioblastoma patient-derived cells through coupling to Gα12/Gαq (Loskutov et al., 2018).

### Multiple Sclerosis (MS) and Experimental Autoimmune Encephalomyelitis (EAE)

The pathogeneses of MS and EAE involve inflammation in the CNS (microglial polarization) and demyelination (oligodendrocyte death and myelin damage). During remyelination, M1 (pro-inflammatory) macrophages phagocytize myelin debris and induce OPCs to proliferate and migrate to the lesion site. Next, a switch from M1 (pro-inflammatory) to M2 (anti-inflammatory) phenotype induces the secretion of trophic factors that promotes the differentiation of OPCs into new mature and myelinating oligodendrocytes (Fransson et al., 2020). Fransson et al. (2020) analyzed *Lpar1*/Edg2 expression levels in peripheral blood mononuclear cells (PBMCs) from EAE mice and patients with relapsing MS and found increased *Lpar1*/Edg2 expression in these cells. The levels of different M1 markers, such as CCL2, CCL20, CCL5, and TLR2, were shown to be increased after incubation with LPA, and M1 polarization was shown to be partially suppressed by the addition of an *Lpar1/*Edg2 inhibitor (Ki16425). Collectively, these results indicate that LPA activates a pro-inflammatory response and triggers M1 polarization through *Lpar1/*Edg2 (Fransson et al., 2020). Gintonin, a ginseng-derived exogenous LPA ligand, may alleviate demyelination and microglial polarization in EAE by enhancing anti-inflammatory and antioxidant activities through stimulating the Lpar1/MAPK and Kelch-like ECH associated protein 1 (Keap1)-nuclear factor erythroid 2-related factor 2 (Nrf2) pathways (Choi et al., 2020).

### White Matter Injury (WMI)

The most common type of brain injury in premature neonates is diffuse WMI, which involves oligodendrocyte maturation arrest and hypomyelination, resulting in cognitive, behavioral, and sensory disabilities as well as psychological disorders later in life (van Tilborg et al., 2016). Garcia-Diaz et al. (2015) found that loss of *Lpar1* suppresses oligodendrocyte differentiation and myelination, suggesting that *Lpar1* plays an important role in WMI. WMI has been frequently reported in HIV-infected patients. It has been reported that ATX-LPA signaling participates in oligodendrocyte differentiation in HIV-infected patients (Wheeler et al., 2016). Collectively, we speculate that *Lpar1*/Edg2 may promote oligodendrocyte differentiation and protect against WMI.

### Ischemia-Reperfusion Injury

Lysophosphatidic acid receptor 1/Endothelial differentiation gene-2 receptor expression is upregulated in the vulnerable inner retinal layers secondary to ischemia-reperfusion injury. Hirata et al. (2007) analyzed the expression of genes and confirmed the results by evaluating protein expression to determine the neuroprotective mechanism of HBO, and the researchers found that *Lpar1*/Edg2 expression is significantly upregulated by HBO treatment. Upregulation of *Lpar1/*Edg2 expression plays an important neuroprotective role upon HBO treatment after ischemic brain injury (Hirata et al., 2007). *Lpar1*/Edg2 can prevent apoptosis of numerous neuronal cell types and glial cells after ischemia-reperfusion injury (Savitz et al., 2006).

### Neuropathic Pain

Lysophosphatidic acid receptor 1/Endothelial differentiation gene-2 receptor is expressed in both DRG neurons and the dorsal root (Nagai et al., 2010). Uchida et al. (2014) found that *Lpar1/*Edg2-mediated amplification of spinal LPA production is required for the induction of neuropathic pain after nerve injury. LPA-*Lpar1* and Rho-ROCK activation was shown to be crucial for the development of allodynia and hyperalgesia in a model of neuropathic pain (Inoue et al., 2004). LPA-induced upregulation of *Lpar1/*Edg2 and Nav1.8 expression in the DRGs of normal rats results in pain behavior in bone cancer (Pan et al., 2016). LPA is important for the initiation of neuropathic pain via the coupling of *Lpar1/*Edg2 to G proteins and downstream propagation of *Lpar1/*Edg2 signals through the MAPK, protein kinase C (PKC), and Rho pathways (Pan et al., 2016). *Lpar1/*Edg2-mediated microglial activation, ATP release and BDNF expression upregulation may contribute to the maintenance of neuropathic pain (Fujita et al., 2008).

### Psychiatric Disorders

The *Lpar1/*Edg2 level in the whole peripheral blood of patients with major depression is related to mood (Kéri et al., 2014). *Lpar1/*Edg2 is also one of the ten top blood candidate biomarker genes for mood (Le-Niculescu et al., 2009). *Lpar1/*Edg2 expression is downregulated in the temporal cortices of subjects with major depressive disorder compared with those of control participants (Aston et al., 2005) and in the peripheral blood lymphocytes of schizophrenia patients compared with those of control participants (Bowden et al., 2006). The *maLpar1-null* mouse model exhibits depressive and anxious behaviors, which implies that researchers should develop drugs targeting *Lpar1/*Edg2 receptor as treatments for depression, mainly the anxious subtype (Moreno-Fernández et al., 2018).

Psychiatric disorders including schizophrenia, affect public health and are thought to have an etiology. The association between Lpar1 and schizophrenia was reported by some studies (Harrison et al., 2003; Roberts et al., 2005). They reported that a marked deficit in prepulse inhibition and alteration of neurotransmitter in Lpar1 (^–^/^–^) mice compared with wild type. Roberts et al. (2005) reported that Lpar1 mutation produces a number of changes in neurotransmitters, including 5-hydroxytryptamine (5-HT), tyrosine, aspartate, glutamate and γ-aminobutyric acid (GABA), that have been associated with a schizophrenia-like pathology in mice model. They found that a significant decrease of 5-HT and GABA, while a significant increase of tyrosine, aspartate and glutamate was observed in *Lpar1* (^–^/^–^) mice compared with *Lpar1* (^+^/^+^) mice (Roberts et al., 2005).

### Other Diseases

Lysophosphatidic acid receptor 1/Endothelial differentiation gene-2 receptor, which is expressed on fibers and is present on reactive astrocytes in the corpus callosum, specifically affects axonal damage following traumatic brain injury (TBI) (Frugier et al., 2011). *Circ-Lpar1* expression is upregulated in Alzheimer’s disease (AD) patients compared with control participants (Li et al., 2020). *Circ-Lpar1* levels can predict the risk of AD with an OR of 2.984 (95% CI, 1.644–5.416). *Circ-Lpar1* might regulate neuronal cell death and neuroinflammation by sponging its target miRNAs, thus elevating the risk for AD. The effects of gintonin against Parkinson’s disease are mediated via *Lpar1* (Choi et al., 2018), and the Lpar1/Edg2 was downregulated in Parkinson’s disease rat model (Yang et al., 2015). It demonstrated that low expression of Edg2 in Parkinson’s disease rat model may result in neuron loss, which contributed to the pathology of Parkinson’s disease. Gintonin can activate Keap1-Nrf2 signaling through the LPA-*Lpar1* signaling pathway to treat Huntington’s disease (HD) (Jang et al., 2019). *Lpar1*/Edg2 is also differentially expressed in the post-mortem brains of cocaine abusers compared to those of control participants (Kristiansen et al., 2009). Additionally, Lpar1 is required for normal development of neonatal behavior. Lpar1 deletion results in impaired suckling in neonatal pups, and the sucking defect may responsible for Lpar1 deletion induced death (Contos J.J.A. et al., 2000), and Lummis et al. (2019) reported that Lpar1 was identified as a key mediator of posthemorrhagic hydrocephalus (PHH) in premature animal model.

## Conclusion and Future Perspectives

Collectively, these findings suggest that *Lpar1* is essential for maintaining the normal functions of the CNS. Any alteration in *Lpar1* function or expression may lead to CNS disorders or diseases. The upstream molecules that regulate Lpar1 in the CNS need to be further studied.

MicroRNAs are short nucleotides that can bind to target mRNAs and then inhibit their translation into proteins. Binding of miR-892b to *Lpar1* mRNA can downregulate Edg2 expression. LncRNAs, as competing RNAs (ceRNAs), can bind to microRNAs to inhibit microRNA function, especially by upregulating target protein expression. It has been reported that lncRNA ZFAS1 enhances tumorigenesis and metastasis in nasopharyngeal carcinoma by upregulating Edg2 expression in a miR-892b-dependent manner (Peng et al., 2020). Additionally, the genes that bind to the promotor of *Lpar1*, such as HEB, might regulate its expression and affect its function. Future studies will further determine whether these upstream molecules regulate Lpar1, especially in the context of demyelinating diseases and postnatal WMI.

A recent study revealed that Lpar1/Edg2 expression is increased in PBMCs from EAE mice and patients with relapsing MS; additionally, circ-Lpar1 expression in upregulated in AD patients compared with control participants. These studies indicate that Lpar1/Edg2 may aid in diagnosis in the near future and be a therapeutic target.

## Author Contributions

DX, XL, and YQ: conceptualization. DX, XS, HG, XL, and YQ: software, validation, investigation, resources, and writing—review and editing. DX: writing—original draft preparation. XL and YQ: visualization and supervision. DX, HG, XL, and YQ: funding acquisition. All authors read and approved the final manuscript.

## Conflict of Interest

The authors declare that the research was conducted in the absence of any commercial or financial relationships that could be construed as a potential conflict of interest.

## Publisher’s Note

All claims expressed in this article are solely those of the authors and do not necessarily represent those of their affiliated organizations, or those of the publisher, the editors and the reviewers. Any product that may be evaluated in this article, or claim that may be made by its manufacturer, is not guaranteed or endorsed by the publisher.

## Abbreviations

AD: Alzheimer’s disease
AJs: adherens junctions
2ccPA: 2-O-carba-cyclic phosphatidic acid
5-HT: 5-hydroxytryptamine
BDNF: brain-derived neurotrophic factor
CNS: central nervous system
ceRNA: competing RNA
EAE: experimental autoimmune encephalomyelitis
EB3: third E-boxes
Edg2: endothelial differentiation gene-2 receptor
DG: dentate gyrus
DRG: dorsal root ganglion
FGFR: fibroblast growth factor receptor
FRS2 α: FGFR substrate 2 α
GA: Golgi apparatus
GABA: γ-aminobutyric acid
GDNF: glial cell line derived neurotrophic factor
GPCRs: G protein-coupled receptors
HBO: hyperbaric oxygen
HD: Huntington’s disease
HEB: HeLa E-Box binding protein
Keap1: Kelch like ECH associated protein 1
LPA: lysophosphatidic acid (1-acyl-sn-glycerol-3-phosphate)
Lpar1: lysophosphatidic acid receptor 1
LE/Lys: late endosomes/lysosomes
lncRNAs: long non-coding RNAs
MAPK: mitogen-activated protein kinase
MBP: myelin basic protein
MOG: myelin oligodendrocyte glycoprotein
MS: multiple sclerosis
MMPs: matrix metalloproteinases
NPCs: neural progenitor cells
NGF: nerve growth factor
Nrf2: nuclear factor erythroid 2-related factor 2
OPCs: oligodendrocyte precursor cells
P2: postnatal day 2
pre-OLs: premyelinating oligodendrocytes
PHH: posthemorrhagic hydrocephalus
PLP: proteolipid protein
RER: rough endoplasmic reticulum
ROS: reactive oxygen species
RE: recycling endosomes
SGZ: sub-granular zone
SNAREs: soluble N-Ethylmaleimide -sensitive factor attachment protein receptors
SGM: somatic genomic mosaicism
TBI: traumatic brain injury
TNF: tumor necrosis factor
V-SVZ: ventricular-sub-ventricular zone
WMI: white matter injury
4E-BP1: 4E-binding protein.
